# Copy number variants in Ebstein anomaly

**DOI:** 10.1371/journal.pone.0188168

**Published:** 2017-12-07

**Authors:** Andreas Giannakou, Robert J. Sicko, Wei Zhang, Paul Romitti, Marilyn L. Browne, Michele Caggana, Lawrence C. Brody, Laura Jelliffe-Pawlowski, Gary M. Shaw, Denise M. Kay, James L. Mills

**Affiliations:** 1 Division of Intramural Population Health Research, Eunice Kennedy Shriver National Institute of Child Health and Human Development, National Institutes of Health, Department of Health and Human Services, Bethesda, Maryland, United States of America; 2 Division of Genetics, Wadsworth Center, New York State Department of Health, Albany, New York, United States of America; 3 Department of Epidemiology, College of Public Health, The University of Iowa, Iowa City, Iowa, United States of America; 4 Department of Epidemiology and Biostatistics, University at Albany School of Public Health, Rensselaer, New York, United States of America; 5 Medical Genomics and Metabolic Genetics Branch, National Human Genome Research Institute, National Institutes of Health, Bethesda, Maryland, United States of America; 6 California University of California San Francisco School of Medicine, San Francisco, California, United States of America; 7 Department of Pediatrics, Stanford University School of Medicine, Stanford, California, United States of America; Rutgers University Newark, UNITED STATES

## Abstract

**Background:**

Ebstein anomaly (EA) is a rare congenital defect characterized by apical displacement of the septal tricuspid leaflets and atrialization of the right ventricle. The etiology of EA is unclear; however, recurrence in families and the association of EA with genetic syndromes and copy number variants (CNVs) suggest a genetic component.

**Objective:**

We performed a population-based study to search for recurrent and novel CNVs in a previously unreported set of EA cases.

**Methods:**

We genotyped 60 EA cases identified from all live births (2,891,076) from selected California counties (1991–2010) using the Illumina HumanOmni2.5–8 array. We identified 38 candidate CNVs in 28 (46%) cases and prioritized and validated 11 CNVs based on the genes included.

**Results:**

Five CNVs (41%) overlapped or were close to genes involved in early myocardial development, including *NODAL*, *PDLIM5*, *SIX1*, *ASF1A* and *FGF12*. We also replicated a previous association of EA with CNVs at 1p34.1 and *AKAP12*. Finally, we identified four CNVs overlapping or in close proximity to the transcription factors *HES3*, *TRIM71*, *CUX1* and *EIF4EBP2*.

**Conclusions:**

This study supports the relationship of genetic factors to EA and demonstrates that defects in cardiomyocytes and myocardium differentiation may play a role. Abnormal differentiation of cardiomyocytes and how genetic factors contribute should be examined for their association with EA.

## Introduction

Ebstein anomaly (EA) is a rare congenital malformation of the tricuspid valve and right ventricle characterized by apical displacement of the origin of septal tricuspid leaflets and atrialization of the right ventricle [1]. Additional cardiac defects such as patent foramen ovale (PFO), atrial septal defect, ventricular septal defect, pulmonary outflow obstruction, patent ductus arteriosus (PDA), accessory conduction pathways, bicuspid aortic valve and left heart lesions like mitral valve prolapse and left ventricular noncompaction have been associated with EA [1–4]. In fact, in 39% of EA cases, a left heart defect has been reported [4]. The estimated prevalence of EA is 1 in 20,000 live births [5–7]. Although diagnosis and treatment for EA have improved greatly, still many symptomatic neonates with EA do not survive beyond the first decade of life [2, 8].

The majority of infants with EA appear to occur sporadically; however, reports of familial recurrence, [9–15] as well as of genetic mutations associated with EA, [16] show evidence of a genetic background. Mutations in sarcomeric protein, myosin heavy chain cardiac muscle beta (*MYH7*) have been linked with EA and cardiomyopathy [17]. EA has also been identified in association with rare mutations, various syndromes, chromosomal abnormalities and copy number variants (CNVs) [18]. In our previous study we linked EA with CNVs, specifically, duplication of 1p34.1 and 8q21.13, genes related to myocardial development, BMP signaling, histone modification and cardiomyocyte differentiation, supporting the involvement of the developing myocardium in the etiology of EA [18].

Although a genetic factor has been identified in a small percentage of EA cases, the number of studies examining genetic factors in association with EA is limited; thus, the genetic components contributing to the development of EA remains largely unclear. In this population-based study, we searched for potentially causal CNVs in EA cases without accompanying major defects.

## Materials and methods

### Ethics statement

The California Department of Public Health (IRB 13-03-1164), the New York State Department of Health (IRB 07–007), and the NIH Office of Human Subjects Research (OHSRP 11631) reviewed and approved this study. Prior to genotyping and analysis, cases were given a random identification number and all personally identifying information was removed.

### Cases

EA cases were identified via the California Birth Defects Monitoring Program (CBDMP), a population-based, active ascertainment birth defects surveillance program. The methods have been described in detail previously [19]. In brief, trained staff collected diagnostic and demographic information. Each diagnosis was coded using a modification of the British Paediatric Association (BPA) Classification of Diseases. Modifications to the BPA codes were developed by the CDC and the CBDMP [19]. A study performed on the CBDMP registry determined that the completeness of ascertainment exceeded 93% [20].

EA cases were identified from all live births (N = 2,891,076) from 1991 to 2002 with maternal residence in San Francisco, Los Angeles, Santa Clara and San Joaquin Valley County (Fresno, Kern, Kings, Madera, Merced, San Joaquin, Stanislaus and Tulare), excluding births at military facilities. Infants with co-occurring syndromes, other major congenital heart defects (CHD) defects or other major non-CHD defects were excluded. Infants with PDA, PFO and bicuspid aortic valve were not excluded from the case group. These exclusions were done to establish a more homogeneous phenotypic EA group for genetic interrogation. A total of 60 cases met the inclusion criteria and we located archived newborn screening dried blood spots (DBS) for the all 60 cases along with eight unaffected live births to serve as controls.

To ensure that the identified CNVs were rare in the unaffected population, we investigated their frequencies in an unaffected group of infants. We randomly selected 165 infants without birth defects derived from the same population (county and year of birth) as the cases, obtained their bloodspots, and performed qPCR copy-number assays using at least one probe per CNV region. Demographic data for all live births in the study counties from 1991 to 2002 were obtained from birth records and compared with the 60 EA cases using Fisher’s exact test or t test.

### DNA extraction

GenSolve DNA recovery kit (GenTegra, Pleasanton, CA) was used to extract DNA from two 3 mm punches from each infant’s dried blood spot. Punches were incubated at 56°C in 620μL freshly prepared GenSolve Recovery Solution A (1% LiDS, Solution A and Proteinase K) with gentle shaking for 1 hour. Following incubation, the blood spots were transferred to a spin basket in a tube containing 20μL of GenSolve Recovery Solution B and centrifuged to collect extracted DNA from the punches. The flow through containing extracted DNA was purified using a QIAamp DNA Mini Kit (QIAGEN, VALENCIA, CA) following the manufacturer's standard protocol. Final elution volume was 50μL.

### CNV detection, selection, and validation

The 60 EA cases, 42 cases of unrelated phenotype, eight controls, (one in duplicate), and one HapMap sample were batched and genotyped. The methods have been described previously [21]. In brief, samples were genotyped using the Illumina HumanOmni2.5-8v1-3_A1 bead arrays and the Infinium LCG assay protocol. The mean sample call rate ± SD (range) was 99.85 ± 0.25 (97.31–99.93). The mean log R ratio deviation was 0.103 ± 0.026 (0.078–0.291). Single nucleotide polymorphism genotype reproducibility was 100% for the duplicated control. Genotype clusters were defined based on the data generated in this project. Genotypes and clusters were manually reviewed and cleaned by re-clustering, editing, and excluding where appropriate. A total of 2,303,118 autosomal markers were included in the CNV analysis. CNVs were called and annotated using pennCNV (version 2011/05/03).

CNVs were excluded if they were shorter than 20 kb, contained fewer than ten SNP probes, overlapped more than 20% with common CNVs in HapMap or CHOP, or overlapped more than 20% with same-type CNVs in an in-house reference CNV database compromised of unaffected controls and cases of other unrelated birth defects. The remaining CNVs were uploaded to DGV (build37/hg19, DGV release date 2016-05-15, and date accessed 2016-12-10) and analyzed for overlap. A CNV was selected for further analysis if it had minimal overlap with variants present in DGV or if the CNV overlapped a gene with no DGV entries significantly overlapping it. Although the DGV is a very valuable resource, some studies in the database have very small sample sizes and all could potentially include false positives. Furthermore, breakpoint determination is not precise. For these reasons, we chose to ignore overlap with low confidence in the DGV browser and overlap with variants from studies using similar methods.

In total, 38 CNVs were considered candidate CNVs for EA. We selected 11 CNVs for validation based on whether they contained biologically relevant genes, or overlapped CNVs that had been previously reported in EA cases. CNV validation studies were performed using one to three quantitative real-time polymerase chain reaction (qPCR) TaqMan assays (Applied Biosystems, Carlsbad, CA, USA) per CNV region. Validations were performed as previously described. [21] All 60 EA cases and four control subjects were included in each assay. We subsequently screened all validated CNVs against an additional 165 control samples from unaffected California live births using at least one assay targeting each area of interest. Therefore, a total of 169 unaffected controls were screened using at least one assay in each candidate CNV region.

## Results

### Demographic data

Of the 2,891,076 live births, 60 met our case definition and inclusion criteria. Maternal age, maternal race/ethnicity or parity did not differ statistically between cases and the general population. Mothers of cases (44.66%) were more likely than mothers of controls (34.16%) to have more than a high school education (*P* = 0.04). Cases had significantly shorter gestation (mean of 268 vs. 274 days; *P* = 0.012). Select demographic characteristics of mothers and EA cases and the California source population are shown in Table 1.

**Table 1:** Select demographic characteristics of EA cases and the California reference population.

Characteristic	Ebstein cases (*n* = 60)	CA live births (*n* = 2,891,076)	*P* value
Maternal age, years (%)			0.80
<20	8 (13.33)	342,386 (11.84)	
20–34	43 (71.66)	2,142,022 (74.09)	
≥35	9 (15.00)	405,980 (14.04)	
Maternal race/ethnicity, n (%)			0.30
Non-Hispanic white	16 (26.66)	705,857 (24.42)	
African American	1 (1.66)	233,872 (8.09)	
Hispanic	34 (56.66)	1,571,379 (54.35)	
Asian	8 (13.33)	340,561 (11.78)	
Other	1 (1.66)	29,404 (1.02)	
Maternal education, years (%)			0.04
<12	23 (38.33)	1,084,417 (37.51)	
12	9 (15.00)	792,778 (27.42)	
>12	28 (46.66)	987,669 (34.16)	
Parity, n (%)			0.90
Nulliparous	24 (40.00)	1,132,626 (39.18)	
Multiparous	36 (60.00)	1,756,322 (60.75)	
Infant sex, n (%)			0.70
Male	29 (48.33)	1,477,612 (51.11)	
Female	31 (51.66)	1,413,423 (48.89)	
Infant gestational age (mean days ± SD)	268 ± 18	274 ± 16	0.012
Infant birth weight (mean grams ± SD)	3195 ± 638	3340 ± 568	0.08

### Genetic analysis

The 60 EA cases genotyped resulted in 2864 PennCNV calls in the microarray analysis. After applying the selection criteria described in the Material and Methods section, we identified 38 CNVs in 28 (46%) cases. We selected 11 CNVs in 10 cases for qPCR confirmation based on the genes included and their functions. All 11 CNVs were validated: seven duplications and four heterozygous deletions ranging from 22 Kb to 331 Kb (Table 2). One validated duplication at 3q28 had overlap with a heterozygous deletion detected in one control, the validated heterozygous deletion at 7q22.1 had overlap with a heterozygous deletion detected in two control samples and the validated deletion at 10q22.1 had overlap with a heterozygous deletion detected in one control sample. Precise breakpoint studies were not performed, so it is unknown whether the CNVs detected in controls are the same size as those detected in our cases.

**Table 2:** Validated CNVs present in individuals with EA.

Locus	Genomic Coordinates (hg19)	Size^a^ (Kb)	Type	Case ID	Candidate Genes/Transcripts^b^
1p34.1	44264435.. 44420754^**c**^	156	DUP	10	*ST3GAL3*, *ARTN*, ***IPO13***, *DPH2*, *ATP6V0B*, *B4GALT2*, *SLC6A9*, *KLF17*
1p36.31	6319898..6347728	27	DUP	6	***HES3***, *GPR153*, *ACOT7*, *ICMT*
3q28^e^	191795892..191986049^d^	190	DUP	5	***FGF12***
3p22.3	32953920..32985486	31	DEL	5	***TRIM71***, *CCR4*, *GLB1*
4q22-4q22.3	95254575.. 95494126	240	DUP	4	*SMARCAD1***, *PDLIM5 (or ENH1)***, *HPGDS*
6q22	119319524..119441955	122	DEL	8	*MCM9***, *ASF1A***, *FAM184A*, *MIR548B*, *MAN1A1*
6q25.1	151334976..151666605	331	DUP	7	*MTHFD1L***, *AKAP12***, *ZBTB2*, *RMND1*, *CCDC170*, *C6orf211*
7q22.1^e^	101671721..101903016	231	DEL	2	***CUX1***, *MIR4285*, *SH2B2*, *SPDYE6*
14q23.1	60961735..61200391	238	DUP	9	*SIX6*, ***SIX1***, *SIX4*
10q22.1^e^	72151028..72173388	22	DEL	1	*LRRC20***, *EIF4EBP2***, ***NODAL***, *PALD1*
22q11.1	17893850..18055605	161	DUP	3	*CECR3*, *CECR2*, *SLC25A18*, *ATP6V1E1*, *BCL2L13*, *BID*

**a** Size and coordinates estimated from array data (pennCNV calls, hg19).

**b** Genes in bold are discussed in the manuscript.

**c** This variation was called as two CNVs by pennCNV. qPCR analysis showed it is likely one large duplication.

**d** PennCNV called this variation as three CNVs. CNV Partition called it as one.

**e** These CNVs had overlap with CNVs identified in controls.

Kb, kilobase pairs; DEL, heterozygous deletion; DUP, heterozygous duplication

Genes implicated in cardiac development were present in five validated CNVs. Four CNVs previously associated with cardiac defects were also identified. Two variants identified in this study, a duplication overlapping *AKAP12* in one case and a duplication at 1p34.1 in another case, had also been identified in cases with EA in our previous study [18].

Four CNVs were identified that overlapped or were close to transcription factors *HES3*, *TRIM71*, *CUX1* and *EIF4EBP2* which are important in cardiac development. *HES3* and *CUX1* have been reported to interact with Notch and Wnt pathways [22, 23] and dysregulation of these signaling pathways is known to lead to congenital heart defects [24, 25]. In case 1 a 22 Kb deletion was identified at 10q22.1, 19 Kb upstream of *NODAL*, which is a factor in cardiac progenitor differentiation [26] and an inducer of *Sox17*, an endoderm-specific gene regulating paracrine signals in cardiogenesis [27]. A 240 Kb duplication intersecting *PDLIM5* was identified at 4q22 in case 4. *PDLIM5* is involved in cardiomyocyte development, differentiation and survival as well as heart development in general [28, 29]. Loss of *PDLIM5* has also been associated with dilated cardiomyopathy [30]. In case 9 a 238 Kb duplication was identified at 14q23.1 encompassing *SIX1*, a gene that is transiently expressed in cardiac progenitor cells and has been reported to function as a regulator of cardiovascular morphogenesis [31]. In case 8 a 122 Kb deletion was identified at 6q22 downstream of *ASF1A*, a factor required for heart development [32]. In case 5 a 190 Kb duplication at 3q28 intersected *FGF12*, a gene that has been suggested to perform embryonic functions during atrial development [33]. *FGF12* is a candidate gene for Brugada syndrome [34] and has been described in association with posterior urethral valves with duplication/triplication in *FGF12* [35]. In case 7 a 331Kb duplication was identified overlapping *AKAP12*, a gene that we have previously linked to EA and cardiomyopathy after identifying a deletion intersecting *AKAP12* in one EA case. [18] A 156 Kb duplication at 1p34.1 was identified in case 10. This duplication overlaps with the 234 Kb duplication at 1p34.1 that we have previously described in association with EA [18]. A 161Kb duplication in case 3 was detected at 22q11, a genetic locus where variations have previously been reported in some congenital heart defect cases [36, 37]. The validated CNVs are described in more detail in Table 2 and the other candidate CNVs that were not selected for validation are reported in Table 3.

**Table 3:** Other candidate CNVs in EA cases.

Locus	Genomic Coordinates (hg19)	Size^a^ (Kb)	Type	Case ID	Candidate Genes/Transcripts
1p35.3	29603999..29649189	45	DUP	11	*PTPRU*
1p31.1	70731885..71028815	2	DUP	12	*LRRC40*, *SRSF11*, *ANKRD13C*, *HHLA3*, *CTH*
1p31.1	71194705..71251704	57	DUP	12	*PTGER3*
1p31.1	75380989..75414243	33	DEL	13	
1p21.3	98953664..99063658	109	DEL	14	
1p21.1	103673437..103715386	41	DUP	15	
1p13.1	116397910..116422557	24	DUP	16	
1q25.1	173205777..173272047	66	DUP	18	*LOC100506023*
1q42.12	225088997..225114987	25	DEL	3	*DNAH14*, *CNIH3*
3q11.2	95215277..95244641	29	DEL	19	
3q26.31	171372590..171419209	46	DUP	20	*PLD1*
4q22.3	97727113..97844528	117	DUP	21	
4q24	104663644..104793185	129	DUP	22	*TACR3*
5q12.3; q13.1	66557363..67070474	513	DUP	23	*MAST4*, *CD180*
5q22.2	111862156..111915902	53	DEL	24	*EPB41L4A-AS2*, *LOC102467214*, *LOC102467216*, *APC*
10p12.31	21621057..21681278	60	DEL	25	*NEBL*, *NEBL-AS1*, *CASC10*, *MIR1915*, *SKIDA1*, *MLLT10*
11p14.2	26824992..26847310	22	DUP	21	*SLC5A12*
11p13	31091431..31513810	422	DUP	26	*DCDC5*, *DCDC1*, *DNAJC24*, *IMMP1L*, *ELP4*
11p11.2	44880092..44938643	58	DUP	27	*TSPAN18*, *TP53I11*,
15q26.2	95405405..95500573	95	DEL	28	*LOC440311*
17q11.1	25532171..25715198	183	DUP	29	*MIR4522*, *WSB1*, *TBC1D3P5*, *KSR1*
19p13.3	1608372..1630678	22	DEL	19	*UQCR11*, *TCF3*
19q13.32	47822138..47845042	23	DUP	5	*C5AR1*, *C5AR2*, *DHX34*
20p12.1	14327758..14352641	25	DEL	30	*MACROD2*, *FLRT3*
20p13	2538422..2618508	80	DEL	19	*TMC2*, *TGM6*, *SNRPB*, *SNORD119*, *ZNF343*, *NOP56*, *MIR1292*, *IDH3B*, *EBF4*, *PCED1A*, *CPXM1*
20p13	4994115..5080382	86	DUP	20	*SLC23A2*, *RASSF2*, *TMEM230*, *PCNA*, *CDS2*

**a** Size and coordinates estimated from array data (pennCNV calls, hg19).

Kb, kilobase pairs; DEL, heterozygous deletion; DUP, heterozygous duplication.

## Discussion

To our knowledge, this is only the second study to perform a genome wide investigation on CNVs in infants with isolated, non-syndromic, EA, following only our previous work. Thus, our study contributes meaningfully to supporting the role of genetic factors in EA. We identified rare, potentially pathogenic CNVs in almost one half of the EA cases. The candidate CNVs that were identified and validated were prioritized because they included genes linked to early heart development and cardiomyocyte differentiation.

In 5 out of 10 cases with rare CNVs, we identified CNVs overlapping, or being in close proximity to, genes linked to early cardiac development including *NODAL*, *PDLIM5*, *SIX1*, *ASF1A* and *FGF12*. Each of these genes plays an important role in cardiomyocyte differentiation, cardiac development and morphogenesis.

*NODAL* is a TGFβ family member and a key factor in cardiac progenitor differentiation [26]. *NODAL* induces *SOX17* which is an endoderm-specific gene that regulates paracrine signals in cardiogenesis [27]. Studies in mice have shown that lack of *Nodal* co-receptor results in failure of embryonic stem (ES) cells to differentiate into cardiomyocytes [38, 39]. *Nodal* has also been shown to activate heart formation in both avian and amphibian embryos [40–46]. In humans, high levels of Activin, a mimic of *NODAL*, have been demonstrated to activate endoderm formation in ES cells [47–50]. Thus, a deletion causing dysregulation of *NODAL* expression could result in defective differentiation of cardiomyocytes and myocardial defects contributing to the cardiac anomaly seen in EA.

*PDLIM5*, also known as Enigma homolog 1 (*ENH1*/*PDLIM5*), is a member of the PDZ-LIM protein group that is highly expressed in the myocardium and plays an important role in differentiation via activation of protein kinases and transcription factors [29]. Specifically, *ENH1* overexpression has been shown to upregulate MyoD and myogenin as well as myogenic transcription factors such as bHLH [29]. Previous studies in mice and C2C12 cells suggest that *ENH1* is involved in cardiomyocyte development and differentiation, heart development in general and embryonic survival [28, 29]. These studies suggest that *ENH1* has an important role in normal development and differentiation of cardiomyocytes; therefore, defective expression could lead to myocardial defects that are associated with EA.

In addition to its importance in development and differentiation, loss of *ENH1* has been linked to dilated cardiomyopathy [30]. The proposed mechanism is destabilization of the ENH-CypherS-Calsarcin protein complex at the Z-line [30]. Recent studies also show that *ENH1*, acting at cardiomyocytes through binding partners, has a pivotal role in the cardiovascular system as a modulator in sustaining contractile activity [51]. All these findings suggest a complex, yet unclear, role of *ENH1* in the developing heart at the level of cardiomyocytes that could potentially explain abnormal myocardial and structural development similar to what is seen in EA.

*SIX1* is a transcription factor that is transiently expressed in cardiac progenitor cells. Together with its canonical coactivator *EYA1*, it is active in mammalian organogenesis [52] and is linked to congenital cardiac abnormalities [53–55]. The *SIX1/EYA1* transcription complex has been suggested to function as a regulator of cardiovascular morphogenesis working as part of the Tbx1-Six1/Eya1-Fgf8 regulatory cascade [31]. *TBX1* and *FGF8* are well established regulators of heart morphogenesis [56, 57]. A variant affecting expression of *SIX1* could result in dysregulation of the Tbx1-Six1/Eya1-Fgf8 regulatory cascade and lead to cardiac structural malformations.

*ASF1A* is a conserved histone chaperone that interacts with histones H3 and H4 [58]. Previous studies have reported the crucial role of histone chaperone activity in normal heart development and our previous work has also underscored the potential role of histone-modifying genes in CHD and EA [18, 32]. *ASF1A* is a member of the HIRA/UBN1/ASF1a complex, a chromatin remodeling complex that interacts with *NKX2-5* and regulates gene expression dynamics in ES cells [32]. *NKX2-5* is a well-established transcription factor, vital for cardiac development and morphogenesis, especially of the right heart [59]. A deletion affecting *ASF1A* could result in an ineffective HIRA/BUN1/ASF1a complex formation that could lead to defective expression of transcription factors and genes necessary for normal heart development.

Finally, *Fgf12* is a gene that is highly expressed in the mouse myocardium, especially the atrium, during embryogenesis [33]. *FGF12* has also been suggested as a candidate gene for Brugada syndrome, a cardiac arrhythmia channelopathy [34].

Our results extend previous reports indicating a genetic component to EA [16–18, 60–62]. Several theories have been proposed to explain the cause of the tricuspid valve abnormality in EA. Suggested mechanisms include failure of the valve to delaminate from the myocardium and abnormal myocardial development or myocardial arrest leading to valve anomalies [2, 63–65].

In our study, 5 out of the 11 validated CNVs identified include genes related to cardiomyocyte development, differentiation and myocardial morphogenesis which may contribute to abnormal right heart development. These findings show that early myocardial development could in fact play a very important role in EA.

**Fig 1:**
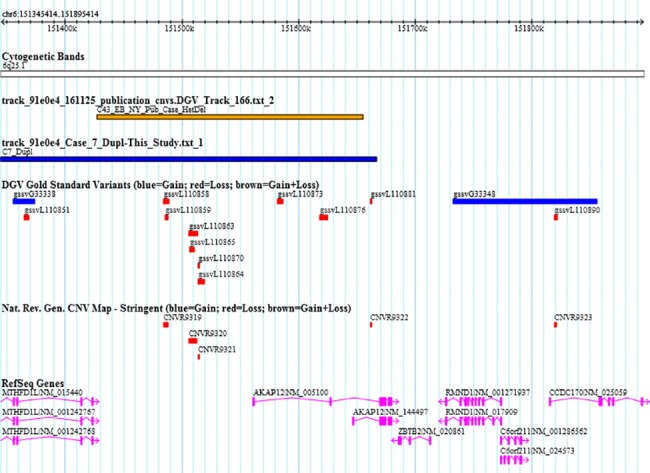
6q25.1 duplication in case 7 (C7_Dupl) overlapping a deletion identified in our previous study (C43_EB_NY_Pub_HetDel).

Interestingly, a 331 Kb duplication was identified in case 7 overlapping *AKAP12*. We have previously identified a deletion in one EA case in this region overlapping the duplication identified in this study (Fig 1) [18]. *AKAP12* (Gravin) is an A-kinase anchoring protein that targets protein kinase A, protein kinase C, calcineurin and other signaling molecules to the beta2-adrenergic receptor. It has been shown that disruption of Gravin leads to augmented contractility and increased baseline cardiac function [66]. In mice homozygous for an *Akap12* mutation, there was increased phosphorylation of Cardiac myosin-binding protein C (cMyBPC) [66] which is a known factor in cardiomyopathy in humans [67].

**Fig 2:**
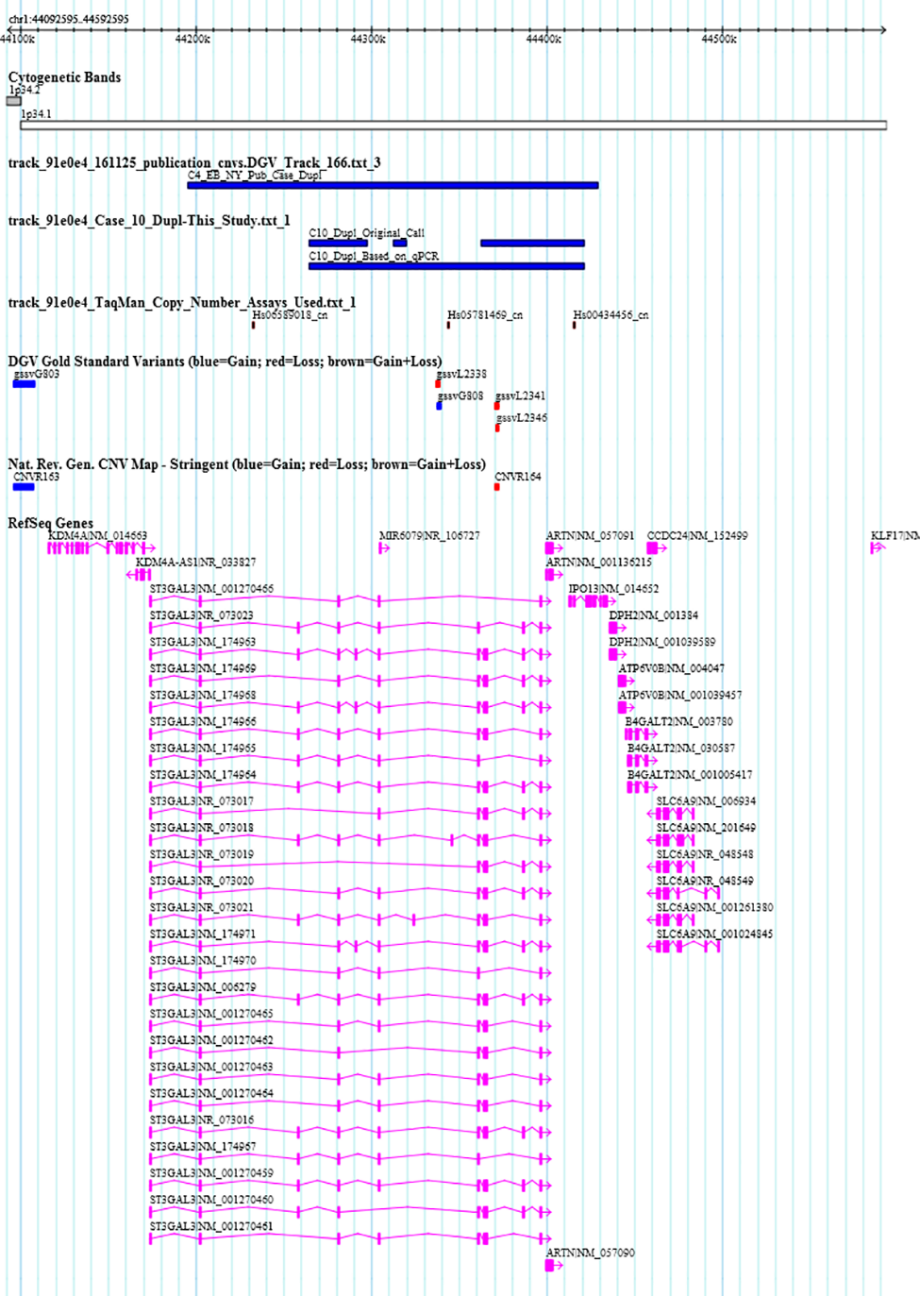
1p34.1 duplication in case 10 (C10_Dupl) overlapping a duplication identified in our previous study (C4_EB_NY_Pub_Case_Dupl).

An important finding of this study is a 156 Kb duplication at 1p34.1 in one CA EA case that overlaps a 234 Kb duplication at the same locus, previously detected in a NY EA case (Fig 2) [18]. Both cases were Black/African American. This finding narrows down the region of interest and the genes overlapped by the intersection of the CNVs at 1p34.1 in both studies include *ST3GAL3*, *ARTN* and *IPO13*. *IPO13*, also known as importin13, is highly expressed in heart tissue and it has been shown to act as a carrier of myopodin [68]. Myopodin is a synaptopodin gene family member that is expressed in heart muscle and has been suggested to function as a regulatory protein in signaling pathways between nucleus and the Z-disc during development [69]. According to the integrated regulation from ENCODE tracks in UCSC, the locus of interest is rich in DNaseI hypersensitivity clusters suggesting that 1p34.1 is a regulatory region functionally related to transcriptional activity [70]. Various transcription factors that act at the duplicated part of 1p34.1 including MAX, C-Fos, YY1, SIX5, FOSL2, EGR-1, and AP-2 gamma, have been suggested to play important roles in development and differentiation. In fact, Ap-2 gamma and SIX5 act during embryogenesis and organogenesis. Replicating the association between duplication of 1p34.1 and EA adds additional evidence of a strong association between this region and EA and suggests that 1p34.1 represents an important locus for follow-up genetic and functional studies.

This study has several strengths and weaknesses. First, it is a population-based study, with a representative set of cases from California live births. The data available through the California Birth Defects Monitoring Program (CBDMP) registry have been collected and coded by well-trained staff and provide excellent and unbiased ascertainment of cases. Another strength is our validation of 11 CNVs ensuring that no false positives are present in our final candidate EA-associated CNV list. Our CNV selection criteria included filtering against the DGV database to assure that the detected CNVs were absent or extremely rare in unaffected subjects. Furthermore, to rule out CNVs specific to our study population, we screened the CNVs in 165 control subjects from the California population. Due to the nature of the CNV validation method (TaqMan copy-number assays), we are unable to determine the exact breakpoints of CNVs. Using one assay per CNV region, we are effectively ruling out the presence of CNVs with breakpoints matching those detected in the cases in the 165 control subjects. The study population included only liveborn infants and thus fetuses with EA that were spontaneously lost or electively terminated were not represented. Such EA cases might have a different genetic etiology. The CNVs identified in EA cases could not be further explored for their origin, i.e., inherited or arose de novo, owing to a lack of data from parents. EA phenotypic presentation and severity can vary considerably, however clinical and treatment data are rarely available from such birth defects registries. Moreover, stratifying based on phenotype and severity, if such data were available, would lead to a very small sample size, inadequate to estimate risk. Our objective was to examine a possible genetic profile in an initial set of EA patients and lay the foundation for larger and more refined investigations.

## Conclusion

Our study identified CNVs affecting crucial signaling pathways for cardiomyocyte differentiation and cell-fate determination. In addition, CNVs were identified in loci that overlap, or are in close proximity to, transcription factors. We also replicated, in an independent population, associations of EA with *AKAP12* as well as with 1p34.1duplication. Abnormal differentiation of cardiomyocytes, *AKAP12* and duplication of 1p34.1 are important areas for future investigation into the etiology of EA.
